# Adding structure to land cover – using fractional cover to study animal habitat use

**DOI:** 10.1186/s40462-014-0026-1

**Published:** 2014-12-25

**Authors:** Mirjana Bevanda, Ned Horning, Bjoern Reineking, Marco Heurich, Martin Wegmann, Joerg Mueller

**Affiliations:** Biogeographical Modelling, Bayreuth Center for Ecology and Environmental Research BayCEER, University of Bayreuth, Universitaetsstr. 30, Bayreuth, 95447 Germany; American Museum for Natural History, Central Park West at 79th Street, New York, 10024-5192 NY USA; Unité de recherche écosystèmes montagnards, Irstea, 2 rue de la Papeterie-BP 76, St-Martin-d’Hères, 38402 France; Department of Remote Sensing, Remote Sensing for Biodiversity Unit, University Wuerzburg, Oswald Kuelpe Weg 86, Wuerzburg, 97074 Germany; Bavarian Forest National Park, Department of Research and Documentation, Freyunger Str. 2, Grafenau, 94481 Germany

**Keywords:** Fractional cover, Remote sensing, Land cover classification, Animal movement, Habitat selection, Mixed model

## Abstract

**Background:**

Linking animal movements to landscape features is critical to identify factors that shape the spatial behaviour of animals. Habitat selection is led by behavioural decisions and is shaped by the environment, therefore the landscape is crucial for the analysis. Land cover classification based on ground survey and remote sensing data sets are an established approach to define landscapes for habitat selection analysis.

We investigate an approach for analysing habitat use using continuous land cover information and spatial metrics. This approach uses a continuous representation of the landscape using percentage cover of a chosen land cover type instead of discrete classes. This approach, fractional cover, captures spatial heterogeneity within classes and is therefore capable to provide a more distinct representation of the landscape. The variation in home range sizes is analysed using fractional cover and spatial metrics in conjunction with mixed effect models on red deer position data in the Bohemian Forest, compared over multiple spatio–temporal scales.

**Results:**

We analysed forest fractional cover and a texture metric within each home range showing that variance of fractional cover values and texture explain much of variation in home range sizes. The results show a hump–shaped relationship, leading to smaller home ranges when forest fractional cover is very homogeneous or highly heterogeneous, while intermediate stages lead to larger home ranges.

**Conclusion:**

The application of continuous land cover information in conjunction with spatial metrics proved to be valuable for the explanation of home-range sizes of red deer.

**Electronic supplementary material:**

The online version of this article (doi:10.1186/s40462-014-0026-1) contains supplementary material, which is available to authorized users.

## Background

Habitat use of animals is assumed to be mainly driven by forage availability and is a complex hierarchical process of behavioural responses and choices [1]. Individuals choose habitat that maximizes resources (e.g. food or shelter) and conditions necessary for survival and reproduction [2], whereas these resources are influenced by temporal and spatial variations of the landscape [3]. Habitat selection is led by behavioural decisions and is shaped by the environment, leading to the observed habitat use [4].

A large majority of animals use certain areas without showing a territorial behaviour, referred to as home range. In contrast to territories, a home range has no defended borders [5]. Home ranges are generally defined as the spatial expression of all behaviours an animal performs in order to survive and reproduce [5]. Since home ranges link individual movement paths to dispersal and population dynamics, understanding why and how home range sizes vary between and among species is a fundamental issue in ecology. The current and prospective availability of large movement data sets and remotely sensed environmental information will allow further detailed analysis [6]. Progress in GPS–sensor receiver technology and satellite telemetry makes it possible to track animals over long time spans with high temporal and spatial resolution and to analyse their habitat requirements and movement paths [7].

By studying variation in home range size and identifying the factors involved in such variation, we can identify how habitat influence individual’s habitat use [2] and therefore the variation in home ranges. A number of factors have been adressed for shaping variation in home range sizes, these include the environmental productivity and the heterogeneity of the landscape [8-10]. Especially the availability of forage is a main driver shaping home range sizes [11]. A common trade–off often faced by many large mammals takes places when open habitats provide the best forage, while closed habitats provide shelter against predators and this may vary with different spatio–temporal scales [12].

Typically in habitat use studies the landscape is represented with a categorical habitat map usually derived from a classification [13,14], while in other studies the landscape is represented only by the dominant habitat type [15,16]. A variety of land cover classifications are routinely produced using remotely sensed data such as MODIS and AVHRR [17].

However, the way the landscape is defined is crucial for the analysis of habitat use. In many studies the landscape is defined in land cover categories, containing classes such as “meadows”, “forest” and “agriculture” [13,15] and it is common sense that different needs of an animal corresponds to different land cover types, for example “forest” as areas for shelter and therefore resting or hiding sites, and “meadows” as areas for forage sites [12].

However, landscapes rarely contain sharp borders between cover types although that is how they are portrayed using a classical land cover classification approach. Moreover information about spatial variation within an *a–priori* defined land cover class is not provided when using a classification. A forest might vary spatially due to different age classes of the trees or small tree fall gaps which increase spatial heterogeneity. This within land cover variation is not captured by categorical maps.

Therefore we use a continuous land cover approach such as fractional cover for the inclusion of spatial variation within classes for our analyses. Fractional cover is a multiscale analysis combined with spatial prediction. This method is related to spectral unmixing methods [18]. The fractional cover image are typically created using a higher resolution land cover classification image to calculate fractional cover training data for lower resolution imagery. For each pixel of the coarse resolution image the percentage coverage for each land cover class within the high resolution is calculated and used for a spatial prediction of the land cover percentages. The percentage cover for the chosen land cover types per pixel of the coarse resolution image is provided as result.

With this approach a continuous land cover classification can be derived which captures the spatial structure in a fine scale manner and this provides a more realistic and more ecologically meaningful representation of the landscape. Global maps with similar approaches of percentage coverage already exist such as MODIS or AVHRR [19,20] however only at a coarse spatial resolution and not validated in the study area.

Furthermore in many habitat use studies forests have structural attributes like “dense forest” or “light forest” with corresponding functional effects, such as light forest with plentiful food resources due to an established understory as enough sunlight can reach the forest floor. However, these structural attributes are often not validated and instead they are implicitly assumed [21]. With the fractional cover approach these structural attributes can be addressed clearly.

In this study, we investigate the potential of continuous land cover information for habitat use of red deer in the Bohemian Forest. As habitat use leads to differing home range sizes, we investigate the potential of continuous land cover information and its spatial representation for the explanation of their variation in size. We hypothesize larger home ranges with increasing forest cover due to lower density of food resources. We test our hypothesis on different spatial (90%, 70% and 50% isopleths) and temporal scales (monthly, biweekly and weekly) to account for temporal and spatial differences.

## Methods

### Study area

The study area is located in Central Europe in the Bohemian Forest, an area belonging to two national parks: the Bavarian Forest National Park on the German side of the border (240 km ^2^) and the Šumava National Park on the Czech Republic side of the border (640 km ^2^). These protected areas are embedded within the Bavarian Forest Nature Park (3070 km ^2^) and the Šumava Landscape Protection Area (1000 km ^2^). In its entirety, the area is known as the Bohemian Forest Ecosystem. The area is mountainous, with a variation in elevation between 600 and 1450 m.a.s.l.. The mean annual temperature varies between 3°C and 6.5°C along higher elevation and ridges. The mean annual precipitation is between 830 and 2230 mm. Within the park, three major forest types exists: above 1100 m: sub–alpine spruce forests with Norway Spruce (*Picea abies* L.) and some Mountain Ash (*Sorbus aucuparia* L.), on the slopes, between 600 and 1100 m elevation, are mixed montane forests with Norway Spruce, White Fir (*Abies alba* MILL.), European Beech (*Fagus sylvatica* L.), and Sycamore Maple (*Acer pseudoplatanus* L.). In the valley bottoms, spruce forests with Norway Spruce, Mountain Ash, and birches (*Betula pendula* ROTH. and *Betula pubescens* EHRH.) [22]. Since the mid–1990s, the forests of the national park have been affected by massive proliferation of the spruce bark beetle (*Ips typographus*). By 2007, this had resulted in the death of mature spruce stands over an area amounting to 5,600 ha [23,24].

### Red deer data

From 2002–2011 red deer were caught during winter, using a procedure approved by the Government of Upper Bavaria, Germany. Red deer were captured and fitted with GPS collars (Vectronic Aerospace, Berlin, Germany) in box traps with side windows after they were lured in with food. Here no immobilization was necessary. A second approach was to tranquillize deer by dart gun where they were attracted by food [25]. We collared 80 deer (39 male, 41 female). Ten individuals were collared two or more times. As animals spend the winter in enclosures, we restricted the analysis temporally from May to the end of September. The most common protocol was to mark red deer in late winter and retrieve the collars after a year by collar drop–off or recapturing, allowing the collars to be used on new individuals. We removed spatial and temporal false fixes (i.e. locations taken only a few seconds apart) beforehand. We defined the samples from the multiple collared animals over the single year as independent. As the schedule of the collars are adjusted to take a location every 15 min for one day of the week we took a random sample of animals with sequences of short time intervals to ensure that all locations have a minimum interval of one hour. The median accuracy of the GPS locations was 16.5 m [26].

### Home range estimation

Home ranges were estimated with a commonly used approach, the fixed kernel method [27,28] using the reference method for the smoothing factor h [29]. We used three different home range definitions to include a spatial scale and to investigate the effect on the core area (50% kernel) and a wider range (70% kernel, 90% kernel). In addition, all home range definitions were estimated on three temporal scales: monthly, biweekly and weekly. We only estimated home ranges for individuals with at least ten locations for a given temporal scale, after removing spatial and temporal outliers [30].

### Representation of the landscape

**Figure 1:**
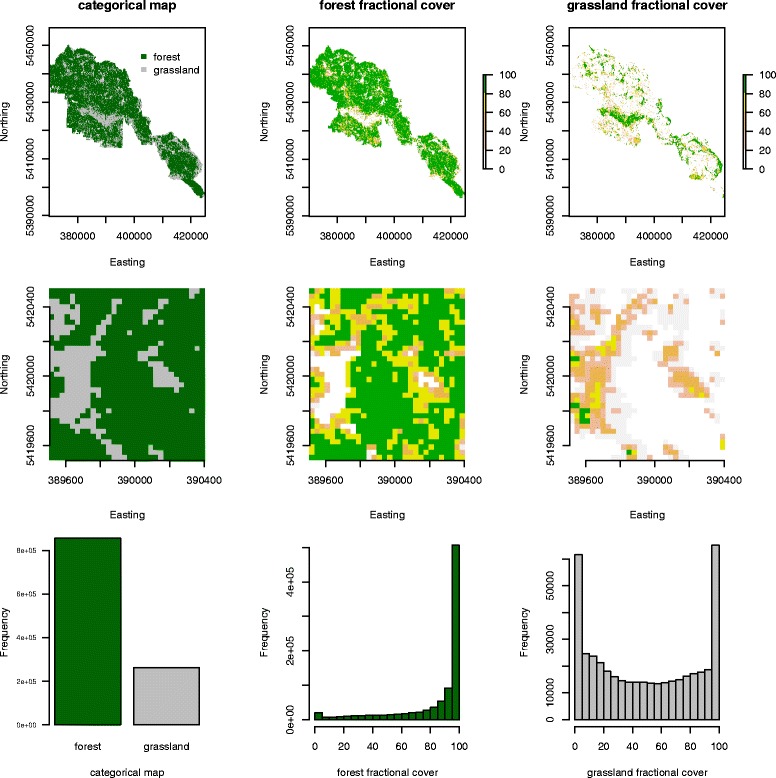
**Overview of the landcover and fractional cover values within the study area.** The upper panels show the distribution of the categorical (left hand side) and continuous fractional cover values (middel and right hand panel). The second row shows a zoom–in for better representation and the last row shows the distribution of the values for the whole study area.

For the calculation of fractional cover a high resolution classified image was derived from aerial images and was used for training. The classified image contained 26 categories (different forest types such as coniferous, deciduous and mixed forest, and age classes such as mature, medium, young). Due to used spatial and spectral resolution we grouped those classes to three major categories in order to be able to discriminate them appropriately: forest (containing all forest types and age classes), open areas (e.g. meadows, regeneration areas, clear cut areas) and others (e.g. water, rocks, roads). To create our training data the fractional cover of each class within 30 m Landsat pixels was calculated. The resulting percent cover values for a particular class were used as response variables to train a random forest (RF) regression model [31]. Random forest uses an ensemble of decision trees (in our case regression trees) to model non-linear relations among response variables [32-34]. The resulting RF model was then used to predict percent cover for the cover type being modelled on a Landsat image using pixel spectral values as predictor variables. The number of regression trees used in the random forest model was 1000, the number of predictors tried on each split was set to the algorithm’s default value (number of Landsat image bands/3). An unbiased accuracy assessment is provided by RF using “Out Of Bag” statistics calculated from a random selection of 1/3 of the training data [31]. Three cloud free Landsat 5 scenes (path 192, row 26) with bands 1–5 from 2006 (July 15th, October 19th) and 2009 (September 9th) were used for the fractional cover analysis. The three predicted vegetation layers complement each other and sum up to 100%. The class “others” contains only small values in our study area, therefore the major part of the values are split between “forest” and “grassland”. Since both layers complement each other we included only the class “forest” in our analysis. Figure 1 shows the categorical map and the fractional cover layers “forest” and “grassland” for the whole study area (upper panels). An enlarged display of a section shows how the formerly categorical representation of the landscape is now split up in continuous values (middle panels). The lower panels show the representation of the categorical values within the fractional cover values in a histogram. The discrete classes are represented by very high cover values within the study area (see Additional file 1: Figure S3 for a figure of the observed vs. predicted values of the regression model).

We extracted all fractional cover values of the forest class within the home ranges and calculated mean, standard deviation and variance. In addition to fractional cover we chose to also calculate texture measures for each home range. Texture metrics were developed by Haralick et al. (1973) [35] and capture habitat structure which can be quantified using the variability of pixel values in a given area. Second–order texture measures are calculated from the gray–level co–occurrence matrix (GLCM) and account for spatial arrangement of pixel values. Haralick et al. (1973) [35] presented a variety of different texture metrics, however he states that these metrics are highly correlated and can be difficult to interpret. To ensure that the chosen texture metric is not size dependent we calculated buffers from 500 to 7000 m in 500 m steps around the home range centres of the 90% kernel isopleths and analysed all texture metrics with regard to their size dependency. We calculated texture measures using all pixel values within the home range. A moving window was used to calculate the texture metric for every pixel relative to its direct neighbours (eight pixels around a centre pixel). We then averaged the resulting texture values to obtain one value for the home range to fit into the mixed model design. We chose to use the texture metric “contrast”, as it shows the least size dependency (see Additional file 1: Figure S1 and is easy to interpret as a measure of local variation in the image and therefore an indicator of landscape heterogeneity. Throughout the remaining text we will refer to the contrast metric as a texture metric or simply as texture.

We choose to use standard deviation of the forest fractional cover calculated within a home range as a measure for variability and the mean forest fractional cover as an estimate of overall forest fractional cover within each home range. Since variables standard deviation and variance show high collinearity [36], variance is not considered in the analysis. For simplicity we will refer to the standard deviation as variation of fractional cover values.

Furthermore we estimated the mean elevation of the home ranges using the 30 m ASTER Global Digital Elevation Map (GDEM) (http://asterweb.jpl.nasa.gov/gdem.asp).

The chosen variables showed no correlation with each other (Pearson’s correlation with the threshold set to 0.7, -0.7 respectively).

### Statistical analysis

To investigate the influence of forest fractional cover and texture on home range sizes, we used linear mixed models [37] on the log transformed home range areas (km ^2^). Afterwards we ran a backfit on the t–values to derive the essential variables [38]. Preliminary analysis showed that the variables texture and elevation have a hump–shaped relationship with home range size in the red deer data and we therefore used a quadratic fit in the models.

Following the framework of Zuur et al. (2009) [39] for mixed effect models, we first identified the best structure for the random effect term. We fitted random intercepts for each individual (ID), different sexes and the year the locations were sampled, using the full model with respect to fixed effects terms and using the REML criterion for fitting. We started with the full random term and then simplified the model. Afterwards we compared the models with an ANOVA and the best model was evaluated with the Akaike Information Criterion (AIC). For variable selection, models were fitted with a maximum likelihood criterion. We considered as fixed effects the mean value of the fractional cover layer forest within a home range, the standard deviation of fractional cover values within a home range, the texture metric contrast and elevation. The final models where fitted using the REML criterion. We derived minimal adequate models by backward stepwise selection using a t–value of 2 as a threshold for inclusion [38]. We repeated the analysis for the three definitions of home range size and for the three definitions of temporal scale.

We used the software tool R version 3.0.1 [40] for all analysis. The package “adehabitatHR” [28] was used for the kernel calculations, “raster” [41], “EBImage” [42] and “randomForest” [43] for creation of the environmental variables and “lmer” [37] and “LMERConvenienceFunctions” [38] were used for the statistical analyses.

## Results

**Figure 2:**
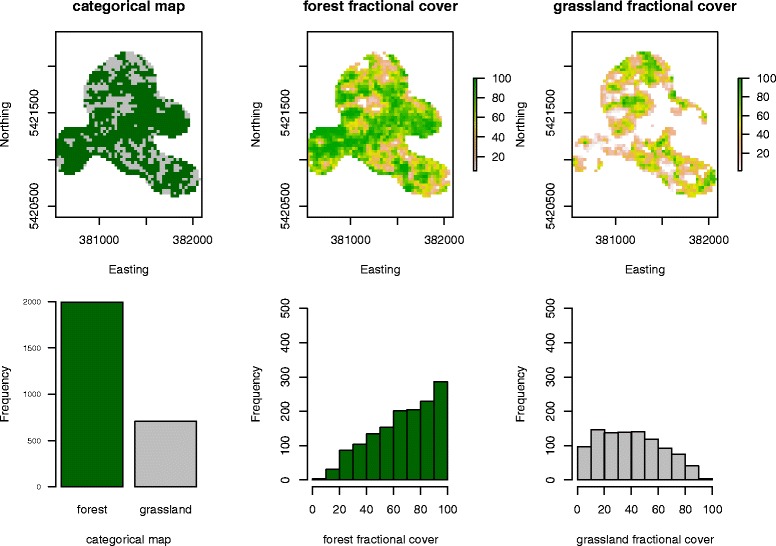
**Representation of the landscape for one home range with both approaches, the categorical and the continuous fractional cover.** The lower panels show the distribution of the values within the home range for each approach.

The fractional cover approach allows a differentiation of variations within land cover types, compared to categorical classes. The spatial heterogeneity of within class variation is captured by this approach. The fit of the random forest regression model for the forest layer was 70.15%. The diversity of fractional cover values within the home range level can be seen in Figure 2. As outlined in Figure 1, the corresponding categorical values are represented by the very high percentage values within the fractional Cover approach.

Home ranges of red deer show a high variation in size in our study area (Additional file 1: Table S1). We analysed the variation of home range sizes with a mixed model, using mean and standard deviation of the forest fractional cover, as well as the variable elevation and a texture metric. The main random effect in all models was the individual effect (variable ID) with an explained deviance of 0.26–0.38% (Additional file 1: Table S3). The fixed effects of the most parsimonious models explained between 26.88% and 30.88% of the observed variation in home range size for red deer across the different spatio–temporal scales (Additional file 1: Table S2).

In all models the texture metric showed the highest explained deviance (7.98%–14.72%) across scales and was the dominant variable explaining variation in home range size with a hump–shaped relationship (Figure S3, Additional file 1: Table S2). However, this hump–shaped relationship was only pronounced at the monthly time scale, whereas in the biweekly and weekly time scale this relationship changed to a negative linear relationship. The texture metric can be interpreted as an index for spatial heterogeneity in a given area. Hence, at larger temporal scales very homogeneous and very heterogeneous landscapes are leading to small home ranges, while at smaller temporal scales only very heterogeneous landscapes lead to small home ranges.

**Figure 3:**
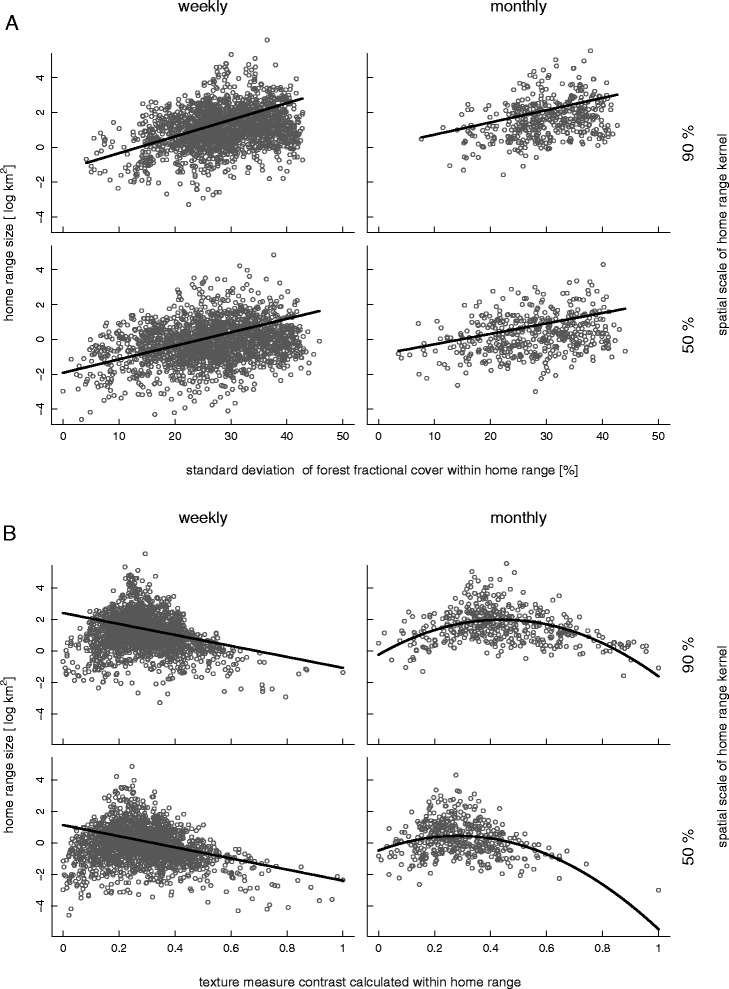
**Plot of log–transformed home range sizes (km** ^**2**^ **) for red deer in relation to (A) the standard deviation of the forest fractional cover values within each home range and (B) the texture measure calculated within each home range.** Home ranges were calculated with the kernel method and the smoothing factor h. Estimates are given for the 90% and 50% kernels and the weekly and monthly time scale. Lines show predicted values and points raw residuals.

Furthermore the variation of forest fractional cover (the standard variation) within a home range contributes significantly with an explained deviance of 7.22–11.59% and a positive relationship, leading to larger home ranges where the variation of forest fractional cover values is higher (Figure 3).

Additionally mean showed a positive effect (5.48–7.12% explained deviance), with no effect on the monthly time scale kernel 50% isopleth (Additional file 1: Figure S2A).

Elevation had a hump–shaped effect on home range size and showed a low explanatory value of 0.35%–6.02% (Additional file 1: Figure S2B).

## Discussion

Many studies of habitat use and home range variation consider the landscape as a categorical map with defined and clearly separated patches [13,14]. This study investigates the use of continuous land cover information, fractional cover, to analyse the within land cover class variation of home ranges over different spatial and temporal scales for red deer in the Bohemian Forest. We demonstrate that small scale variations represented by continuous landscape data provide important information for modelling habitat use.

Red deer as a mixed feeder [44] has the ability to digest a broad spectrum of food items and benefits from forest edges and from the food supply of younger forest stands which show a low forest canopy cover and therefore have a pronounced understory, as sunlight can reach the ground. Mean forest fractional cover shows a positive relationship with home range size meaning that a higher proportion of dense forest will lead to larger home ranges. Whereas in forest patches with less crown cover and therefore more heterogeneous structure, food resources are more abundant which leads to smaller home ranges. This result is in support with other studies [14,45,46]. Mean forest fractional cover is a rather unsuitable derivative, as it averages all pixels within the home range. Nevertheless it shows a significant explanatory value and gives an overview of the overall forest structure within the home range.

The standard deviation of forest fractional cover values captures the variability of values within a home range. High values indicate a wide spectrum of forest fractional cover and therefore a more heterogeneous landscape while small values indicate a more homogeneous landscape within the home range. Tufto et al. (1996) [11] have shown, that female roe deer adjust the size of their home range in response to food supply. In accordance to this study red deer home range sizes increase in our study area with increasing standard deviation and therefore with more heterogeneous forest fractional cover, leading to a higher amount of unfavourable forest habitat within the home range.

The explanatory deviance is largest for the texture metric and also consistent over all spatio–temporal scales with a hump–shaped relationship at larger time scales. Low values of the texture metric correspond to high heterogeneity within the home range, while high values of the texture metric correspond to landscapes which have large aggregated patches. This relationship was detected in a previous study [47] and can be explained by the characteristics of the National Parks. Bark beetle outbreaks in the 90ies affected an area of approximately 5,600 ha especially in the subalpine regions, leading to sunny openings and large regeneration areas characterized by high grass cover, lying dead wood and regrowing vegetation [23]. These areas appear very homogeneous when calculated with a texture metric but offer good habitat for deer, as different resources are provided in a small area, leading to small home ranges, as both requirements, food and cover, are fulfilled at the same spot. Furthermore a heterogeneous landscape, providing many different resources, leads to small home ranges as all the resources needed can be reached within a small distance. The hump–shaped effect flattens in the biweekly and weekly time scale and can only be described with a negative linear trend. However, a pattern towards a hump–shaped distribution can be seen (Figure 3B). This result shows that the temporal scale needs to be accounted for when analysing home ranges as they are likely to change not based on ecological patterns only but on the time scale of the study. The time period of the study is restricted to the summer months, therefore the resource cover can be regarded as static, i.e. not highly changing over the time, while the resource food is dynamic and depleting. Therefore food supply is the main force shaping home range size during summer. When large patches of dense forest occur within the home range, the texture value will increase. These areas provide shelter against predators, but provide only little food resources. Therefore, as food resources are regarded to be a main force shaping home range size, home ranges will increase in size with the inclusion of large patches of dense forest (intermediate values of texture). Furthermore, these regeneration areas are located at higher altitude and are therefore explaining the effect of elevation, reflecting the importance of bark beetle areas in this study. Like the regeneration areas, elevation shows a hump–shaped fit leading to smaller home ranges where important resources are abundant [48].

It is known that other factors, like body mass, age, reproductive status or climatic parameters like temperature or rainfall have an effect on home range size (please see [46] for a more complete list) and it is likely, that by including these parameters, the explanatory value of the models could be increased. However, the best method to estimate home ranges is under debate. While we used at least 10 relocation points [30] to estimate our home ranges other studies suggest at least 20 relocation points [29].

The choice of environmental parameters is important for habitat use modelling. Using classified land cover requires clear definitions of the land cover types but definitions often vary between different maps making them difficult to compare [49]. Moreover do these classes need to reflect the ecological requirements. An increased discrimination of different land cover types is often helpful to better describe a landscape but an increase in the number of land cover classes often results in lower per–class accuracy. Using alternative information such as continuous cover can help to improve how a landscape is represented in a model. Applying remote sensing time–series data can be valuable to further discriminate land cover types and hence allow more fractional cover classes if distinct temporal signature exist for the different targeted land cover types. Applying continuous land cover information for environmental analysis provides detailed information about ecotones and within land cover variation. This research illustrates that fractional cover mapping has potential benefits for ecological research by avoiding categorical values or sharp, most often artificial, boundaries in the landscape. However, the fractional cover approach requires more analytical steps including spatial prediction models and might therefore be potentially biased by the model used.

## Conclusion

The study demonstrates that continuous land cover information can provide valuable information about spatial within class variation as well as gradual vegetation changes, a feature that is not available when using discrete classes. This is especially relevant in movement ecology where a continuous representation of the landscape might be more ecological appropriate. However, to evaluate the added value of the fractional cover approach with regard to land cover classification or biophysical parameter further analysis are needed. Fractional cover mapping of different land cover types adds information, critical to ecological studies, beyond what traditional land cover categorical mapping can offer. As the synergy between remote sensing and ecology increases improved processing and analysis methods will continue to be developed which will have a positive impact on ecological research. These benefits will be especially important with the growing interest in spatio–temporal movement pattern.

## Additional file

Additional file 1
**Additional file contains the: Overview of size dependency of the texture metrics; Overview of red deer home range sizes across spatio–temporal scales; Overview of fixed effects across spatio–temporal scales; Overview of random effect values; Plot of mean forest fractional cover values within home ranges across spatio–temporal scales; Plot of observed and predicted values of the forest fractional cover regression model.**
