# Supplementary material for: Adding structure to land cover – using fractional cover to study animal habitat use
Source: Mov Ecol. 2014 Dec 25;2(1):26. doi: 10.1186/s40462-014-0026-1 (PMC4337748; doi:10.1186/s40462-014-0026-1)
Supplement: Additional file 1 — Additional file contains the: Overview of size dependency of the texture metrics; Overview of red deer home range sizes across spatio–temporal scales; Overview of fixed effects across spatio–temporal scales; Overview of random effect values; Plot of mean forest fractional cover values within home ranges across spatio–temporal scales; Plot of observed and predicted values of the forest fractional cover regression model. [file 40462_2014_26_MOESM1_ESM.pdf]

Additional file

Overview of size dependency of the texture metrics

FIGURE 1: Overview of the sizes dependencies of the texture metrics established by Haralick *et al.* (1973) analysed with a mixed model [2, 3, 4, 5]. Buffers around 90 % kernel home range centres (monthly scale) were drawn from 500 m to 7000 m in 500 m steps around the home range centre for the red deer data set. Buffer index values belonging to the same home range centre point are connected with a line. The explanatory value (expl.dev(%)) of the size dependency for each landscape index is drawn within the plot.

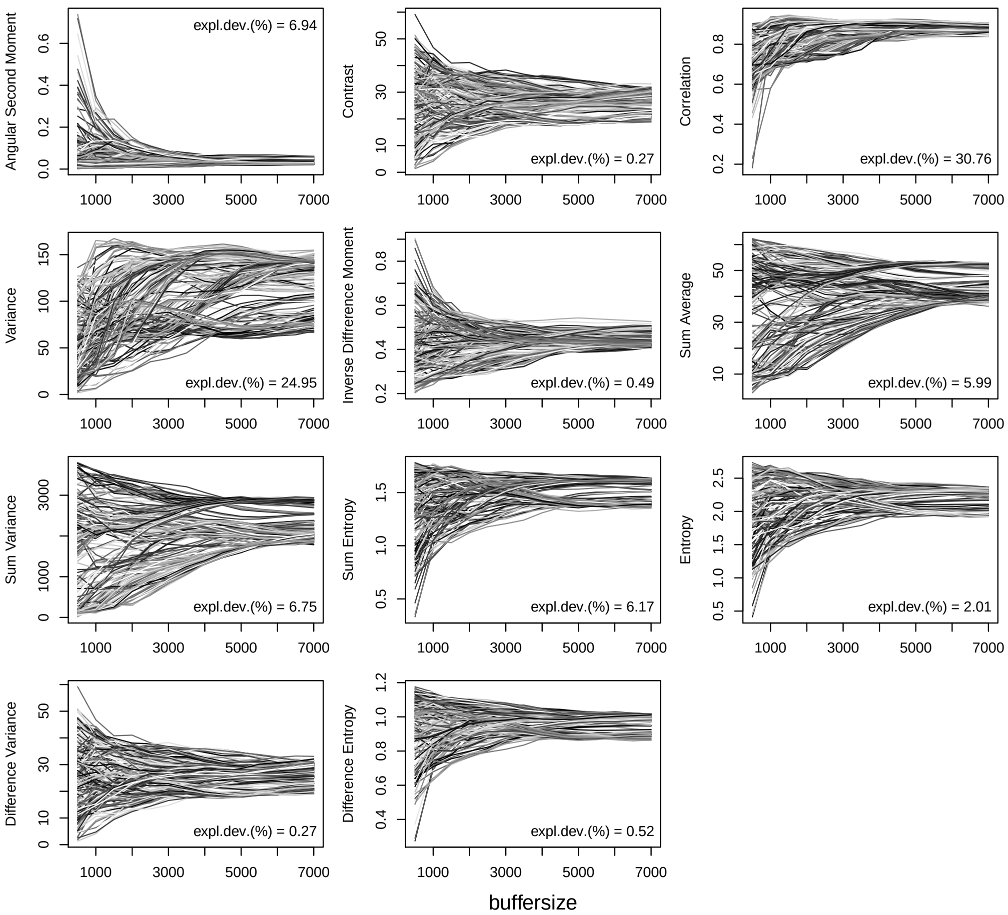

Overiew of red deer home range sizes across spatio–temporal scales

TABLE 1: Summary statistics of red deer home range sizes (km^2) across spatio–temporal scales. N = Number of samples included in home range estimation.

**Red deer (*Cervus elaphus*)**				
		**Kernelsize**		
**Timescale**		**90 %**	**70 %**	**50 %**
**monthly**				
	Mean (range)	9.14 (0.21–142.90)	4.87 (0.10–130.55)	2.60 (0.05–73.76)
	N	456	458	458
**biweekly**				
	Mean (range)	7.15 (0.05–133.41)	3.77 (0.02–90.50)	2.06 (0.01–42.29)
	N	1063	1065	1065
**weekly**				
	Mean (range)	5.78 (0.03–122.84)	3.07 (0.01–115.26)	1.76 (0.01–128.03)
	N	2009	2011	2012

Overview of fixed effects across spatio–temporal scales

TABLE 2: F-values and explained deviance (%) are shown retained from the mixed model calculated for all temporal (monthly, biweekly and weekly) and all spatial scales (90 %, 70 % and 50 % isopleths) with ID as random structure. The variables variance, texture and elevation were fitted as quadratic term.

**Time-scale**	**Kernel-size**	**Variables**	**Estimate**	**Std. Error**	**p–value**	**F–value**	**relation- ship**	**expl.dev. (%)**
weekly	50	mean value	0.008	0.001	< 0.001	215.49	positive	5.48
	50	standard deviation	0.077	0.003	< 0.001	283.61	positive	7.22
	50	texture metric	-3.52	0.64	< 0.001	546.8	negative	13.91
	50	texture metric	-1.69	0.76	0.03	5.99	quadratic	0.15
	50	elevation	< 0.001	< 0.001	< 0.001	25.38	quadratic	0.65
	70	mean value	0.01	0.001	< 0.001	262.67	positive	6.46
	70	standard deviation	0.09	0.004	< 0.001	308.65	positive	7.59
	70	texture metric	-5.49	0.24	< 0.001	585.17	negative	14.38
	70	elevation	< 0.001	< 0.001	< 0.001	14.35	quadratic	0.35
	90	mean value	0.01	0.002	< 0.001	298.05	positive	7.00
	90	standard deviation	0.10	0.004	< 0.001	417.16	positive	9.8
	90	texture metric	-3.39	0.79	< 0.001	565.87	negative	13.3
	90	texture metric	-3.09	0.98	< 0.001	8.95	quadratic	0.21
	90	elevation	0.01	0.002	< 0.001	2.25	positive	0.05
	90	elevation	< 0.001	< 0.001	< 0.001	12.7	quadratic	0.3

Continued on next page

Time-scale	Kernel-size	Variables	Estimate	Std. Error	p–value	F–value	relation- ship	expl.dev. (%)
biweekly	50	mean value	0.008	0l002	< 0.001	135.95	positive	6.37
	50	standard deviation	0.07	0.004	< 0.001	108.88	positive	5.1
	50	texture metric	-5.23	0.32	< 0.001	314.09	negative	14.72
	50	elevation	0.017	0.003	< 0.001	0.13	positive	0.01
	50	elevation	< 0.001	< 0.001	< 0.001	23.83	quadratic	1.12
	70	mean value	0.016	0.002	< 0.001	154.99	positive	7.13
	70	standard deviation	0.08	0.005	< 0.001	117.81	positive	5.42
	70	texture metric	-2.99	0.95	< 0.001	317.24	negative	14.58
	70	texture metric	-2.31	1.06	< 0.001	4.81	quadratic	0.22
	70	elevation		< 0.001	< 0.001	14.46	quadratic	0.66
	90	mean value	0.02	0.002	< 0.001	155.44	positive	6.76
	90	standard deviation	0.09	0.006	< 0.001	194.15	positive	8.44
	90	texture metric	-1.96	0.98	< 0.001	314.23	negative	13.66
	90	texture metric	-3.61	1.11	< 0.001	10.75	quadratic	0.47
	90	elevation	< 0.001	< 0.001	< 0.001	12.32	quadratic	0.54

Continued on next page

Time-scale	Kernel-size	Variables	Estimate	Std. Error	p–value	F–value	relation- ship	expl.dev. (%)
monthly	50	standard deviation	0.07	0.006	< 0.001	74.18	positive	7.66
	50	texture metric	-4.38	0.46	< 0.001	117.77	quadratic	12.16
	50	elevation	0.03	0.004	< 0.001	10.05	positive	1.04
	50	elevation	< 0.001	< 0.001	< 0.001	58.29	quadratic	6.02
	70	mean value	0.009	0.003	0.02	55.84	positive	5.75
	70	standard deviation	0.06	0.008	< 0.001	86.64	positive	8.92
	70	texture metric	-3.84	1.41	< 0.01	101.42	negative	10.44
	70	texture metric	0.13	1.30	0.9	7.99	quadratic	0.82
	70	elevation	0.02	0.006	< 0.001	2.31	positive	0.24
	70	elevation	< 0.001	< 0.001	< 0.001	12.76	quadratic	1.31
	90	mean value	0.01	0.004	< 0.01 0	67.33	positive	6.52
	90	standard deviation	0.07	0.009	< 0.001	119.7	positive	11.59
	90	texture metric	2.04	1.22	0.09	82.4	negative	7.98
	90	texture metric	-4.43	1.17	< 0.001	18.22	quadratic	1.76
	90	elevation	0.02	0.005	< 0.001	2.2	positive	0.21
	90	elevation	< 0.001	< 0.001	< 0.001	9.38	quadratic	0.91

Overview of random effect values

TABLE 3: Table of random effects and standard deviation (SD) of linear mixed models for all spatio–temporal scales for the red deer data set.

**Red deer (*Cervus elaphus*)**				
		**Kernel size**		
**Timescale**		**90 %**	**70 %**	**50 %**
**monthly**				
	random effect	0.28	0.26	0.28
	SD	0.52	0.51	0.53
**biweekly**				
	random effect	0.32	0.28	0.38
	SD	0.57	0.54	0.53
**weekly**				
	random effect	0.38	0.34	0.30
	SD	0.61	0.58	0.55

Plot of elevation and mean forest fractional cover values within home ranges across spatio–temporal scales

FIGURE 2: Plot of log–transformed home range sizes (km^2) for red deer in relation to (A) the mean values of the forest fractional cover values within each home range and (B) the altitude of the home range centres. Home ranges were calculated with the kernel method and the smoothing factor h. Estimates are given for the 90 %, 70 % and 50 % kernels and the weekly, biweekly and monthly time scale. Lines show predicted values and points raw residuals.

A

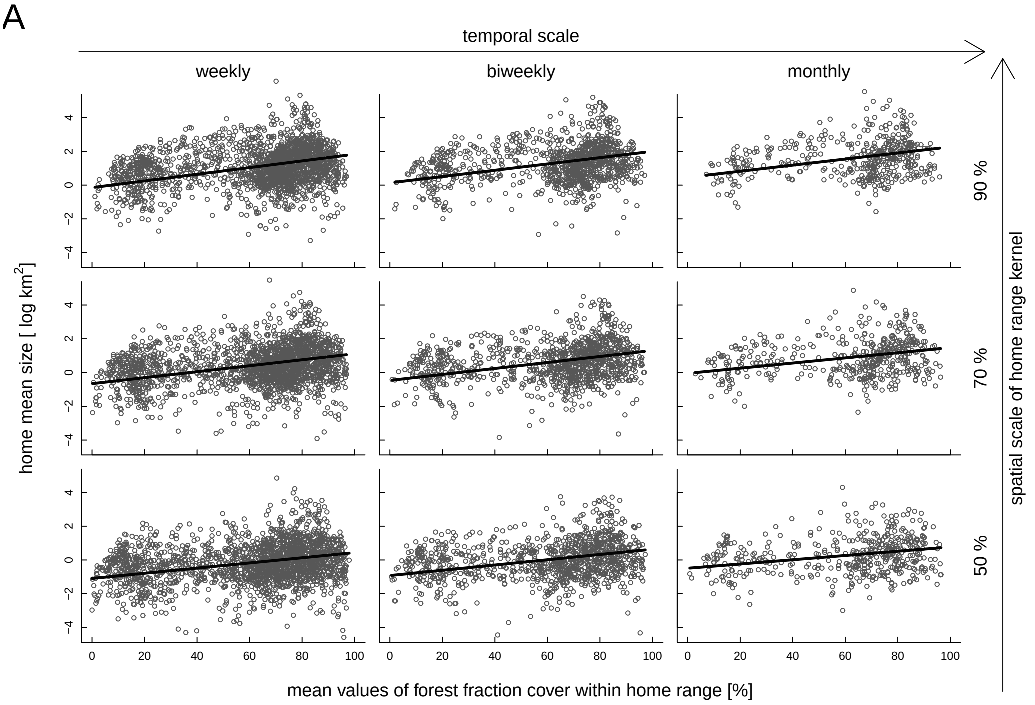

B

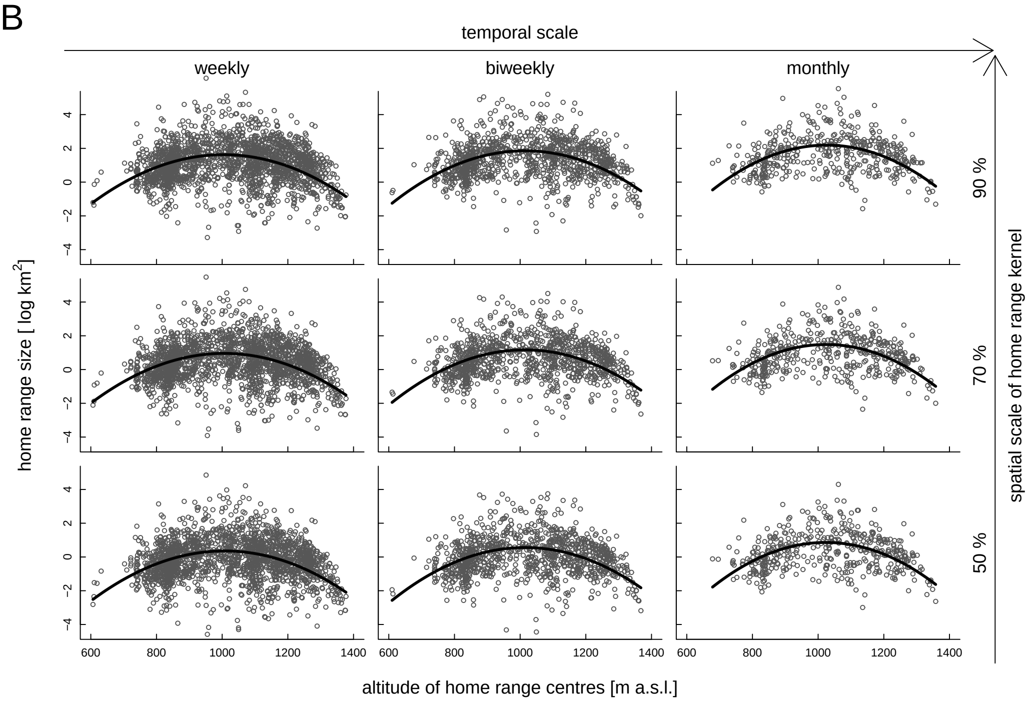
temporal scale
weekly
biweekly
monthly
spatial scale of home range kernel
90 %
70 %
50 %
home range size [log km^2]
altitude of home range centres [m a.s.l.]
600
800
1000
1200
1400
4
2
0
−2
−4

Plot of observed and predicted values of the forest fractional cover regression model

FIGURE 3: This plot shows the fit of the observed vs. predicted values of the forest fractional cover regression model.

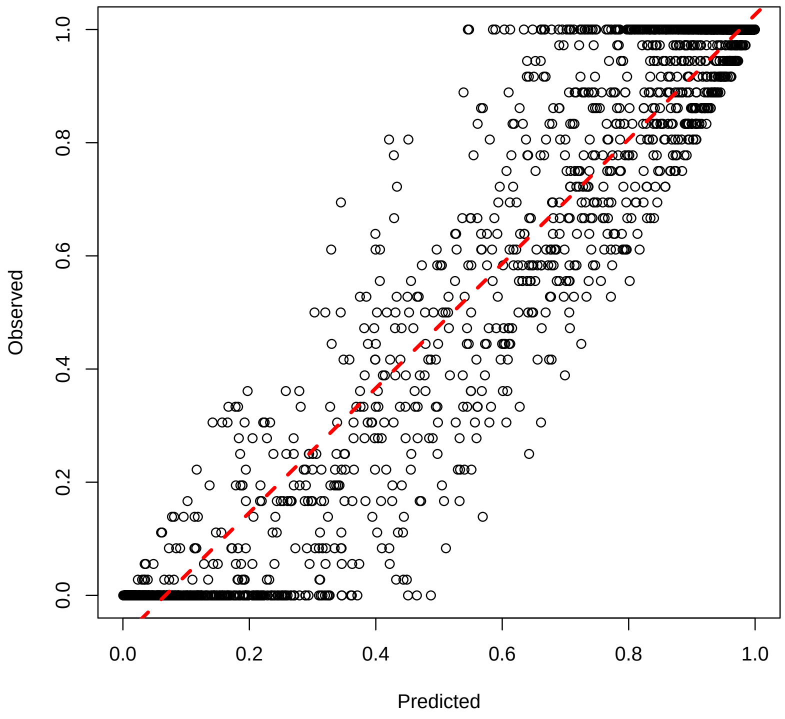

Literature Cited

[1] Haralick, R., Shanmugam, K., Dinstein, I.: Textural features for image classification. IEEE Transactions on Systems, Man and Cybernetics **3**, 610–621 (1973)

[2] Zuur, A.F., Ieno, E.N., Walker, N.J., Saveliev, A.A., Smith, G.M.: Mixed Effects Models and Extensions in Ecology with R, p. 596. Springer, (2009)

[3] R Development Core Team: R: A Language and Environment for Statistical Computing. R Foundation for Statistical Computing, (2013)

[4] Bates, D., Maechler, M., Bolker, B.: lme4: Linear mixed-effects models using S4 classes. R package version 0.9 (2011)

[5] Tremblay, A., Ransijn, J.: LMERConvenienceFunctions: A suite of functions to back-fit fixed effects and forward-fit random effects, as well as other miscellaneous functions. R package version 1.6.8.3 (2011)
